# Imaging Exocytosis in Retinal Bipolar Cells with TIRF Microscopy

**DOI:** 10.3791/1305

**Published:** 2009-06-09

**Authors:** Christina Joselevitch, David Zenisek

**Affiliations:** Cellular and Molecular Physiology, Yale University School of Medicine

## Abstract

Total internal reflectance fluorescence (TIRF) microscopy is a technique that allows the study of events happening at the cell membrane, by selective imaging of fluorescent molecules that are closest to a high refractive index substance such as glass^1^. In this article, we apply this technique to image exocytosis of synaptic vesicles in retinal bipolar cells isolated from the goldfish retina. These neurons are very suitable for this kind of study due to their large axon terminals. By simultaneously patch clamping the bipolar cells, it is possible to investigate the relationship between pre-synaptic voltage and synaptic release^2,3^. Synaptic vesicles inside the bipolar cell terminals are loaded with a fluorescent dye (FM 1-43®) by co-puffing the dye and a ringer solution containing a high K^+^ concentration onto the synaptic terminals. This depolarizes the cells and stimulates endocytosis and consequent dye uptake into the glutamatergic vesicles. After washing the excess dye away for around 30 minutes, cells are ready for being patch clamped and imaged simultaneously with a 488 nm laser. The patch pipette solution contains a rhodamine-based peptide that binds selectively to the synaptic ribbon protein RIBEYE^4^, thereby labeling ribbons specifically when terminals are imaged with a 561 nm laser. This allows the precise localization of active zones and the separation of synaptic from extra-synaptic events.

**Figure:**
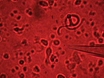


## Protocol

### Part 1: Dissection and Bipolar Cell Isolation

Prepare solutions listed in Table 2; The pH of ringers’ (external) solutions should be adjusted to 7.4 with NaOH and the pH of the internal solution should be adjusted to 7.2 with CsOH. Protect internal solution from light with aluminum foil and keep it at 4 °C until use;
Dark-adapt a goldfish for at least 30 minutes prior to dissection;
While the animal dark-adapts, prepare 5 mL of the hyaluronidase (type V hyaluronidase,1100 units/mL in low Ca^2+^ ringer’s; Sigma, St. Louis, MO) and 10 mL of the L-cysteine (0.5 mg/mL in low Ca^2+^ ringer’s) solutions and weigh the papain (lyophilized powder, 40 units/mL; Sigma, St. Louis, MO) for 5 mL of the digestion solution;
Euthanize the goldfish by quick decapitation with surgical scissors and destroy the brain and spinal cord with a #11 scalpel blade;
Remove the eyes by destroying the extra-ocular muscles with the help of a #7 curved Dumont forceps and cutting the optic nerve with iris scissors;
Place one eye bulb on a piece of filter paper and puncture the scleral limbus with the tip of a #11 scalpel blade;
Introduce the blade of a pair of vannas scissors inside the punctured whole and cut the whole anterior segment away;
Place a small piece of filter paper on top of the remaining optic cup and exert some pressure in order to have the paper soak with vitreous humor;
Lift the filter paper with the retina attached to it and cut the optic nerve with a par of vannas scissors;
Place the filter paper containing the retina in a 35 mm plastic culture dish with hyaluronidase solution and peel off the retina from the filter paper with the help of #7 Dumont tweezers;
Cut the retina into 4-6 pieces with half of an industrial carbon steel single-edged blade and let it sit in the hyaluronidase solution for 20 minutes;
While waiting for hyaluronidase to take effect, add 5 mL of the L-cysteine solution to the papain and let it sit until the liquid becomes transparent (approximately 5-10 minutes);
Wash the pieces of retina 3x in low Ca^2+^ Ringer’s and let them sit in the papain solution for 30-35 minutes;
Wash the pieces of retina 3x in low Ca^2+^ Ringer’s and store them until use at 4 °C in a 35 mm plastic culture dish containing low Ca^2+^ Ringer’s;
To dissociate the cells, put a piece of retina in a microcentrifuge tube containing 500 mL of low Ca^2+^ Ringer’s and slowly triturate the retina by pipetting it up and down with a glass dissociation pipette, carefully not to produce any air bubbles. Dissociation pipettes are fabricated by heating up the tip of a glass Pasteur pipette with a Bunsen burner and slightly bending it with the help of anatomical forceps;
Plate the isolated cells by adding a drop of the retinal suspension to a home-made recording chamber previously filled with 2 mL of low Ca^2+^ ringer’s. The chamber consists of the bottom half of a 35-mm plastic culture dish with a circular whole in the middle and a circular coverslip of 1.78 refractive index glass (PlanOptik, Germany) glued to the bottom with a silicon elastomer (Sylgard 184; Dow Corning, Midland, MI).

### Part 2: Bipolar Cell Loading and Wash Out

TIRF imaging of synaptic vesicles is best carried out using an objective-type TIRFM microscope with a very high NA objective and a sensitive camera. For our experiments, we choose to use a 1.65 NA objective (Apo x100 O HR, N.A. 1.65, Olympus, Japan) with an EMCCD (Cascade 512B, Roper Scientific, Tucson, AZ). The use of the very high NA objective necessitates the use of high refractive glass coverslips and immersion fluid (di-iodomethane with sulfur). Under our conditions, excitation light is limited to an exponentially decaying field with a length constant of approximately 50 nm.

Add a drop of high refractive index liquid (M series, refractive index = 1.7800, Cargille Labs, Cedar Grove, NJ) to the microscope objective;
Place the recording chamber carefully on top of the microscope objective and carefully mount ground electrode and superfusion exit pipe to chamber;
Let chamber sit on microscope for 10-20 minutes to allow cells to sink and adhere to the bottom;
In the meantime, prepare 5 mL of a 1mM trolox® ((±)-6-Hydroxy-2,5,7,8-tetramethylchromane-2-carboxylic acid; Sigma, St. Louis, MO) solution in high K^+^ ringer’s. Sonicate until dissolved;
Prepare 15 mL of ADVASEP-7 washing solution: 1 mM ADVASEP-7 (Sigma, St. Louis, MO) in low Ca^2+^ ringer’s. Note that ADVASEP-7 use is optional and can be omitted if desired;
Purge the superfusion lines and add ADVASEP-7 washing solution, low Ca^2+^ ringer’s and control ringer’s to the superfusion system;
Pull loading pipettes from thin-walled borosilicate glass (Kwik-Fil® TW150-3; WPI, Sarasota, FL). Puffer pipette resistances are in the 1.5-2.5 MΩ range;
Prepare the FM1-43® (N-(3-triethylammoniumpropyl)-4-(4-(dibutylamino)styryl)pyridinium dibromide, “special packaging”; Invitrogen, Carlsbad, CA) solutions. First, make a 1 mM stock by adding 160 μL distilled water to one vial (100 mg) of FM1-43®. This stock can be kept at 4 °C for up to one week. Then, add 5 μL of FM1-43® to 1 mL high K^+^ ringer’s + 1mM trolox®. Protect solution from light with aluminum foil and keep it at 4 °C until use;
Turn the microscope bright field light on and search for intact bipolar cells. Slightly tap the microscope to make sure that the neurons are firmly attached to the bottom of the chamber;
Position the superfusion pen close to the cell of interest and continuously perfuse the preparation with low Ca^2+^ ringer’s;
Turn off the room lights and add a red long pass filter (i.e. RG630; Schott, Germany) to the optical path to minimize excitation of the FM dye;
Fill a loading pipette with 10 μL of the FM dye solution, mount the pipette in the micromanipulator and lower the pipette onto the preparation without overpressure until it is at the same focal plane as the bipolar cell you want to load. Make sure you have at least two electrode holders: the one used for the FM dye cannot be used for patch clamping, or else it may contaminate the intracellular solution;
Position the puffer opening at a distance of around 10 μm from the axon terminal, turn the superfusion system off and puff the dye solution for 10 seconds by turning the pipette overpressure on;
Turn the overpressure off and, without moving the pipette, wait for 30 seconds;
Turn the superfusion on and bathe the chamber in ADVASEP-7 solution for 5 minutes. In the meantime, remove the puffer pipette from the bath;
After 5 minutes, switch to low Ca^2+^ ringer’s and perfuse the chamber for 25-30 minutes to allow removal of excess dye.

### Part 3: Patch Clamping and TIRFM Imaging

While the preparation is washing out, pull patch pipettes from thick-walled borosilicate glass (B150-86-10; Sutter Instrument Company, Novato, CA). Patch pipette resistances are in the 8-10 MΩ range;
After wash out is complete, place the axon terminal in the center of the field of view;
Fill a patch pipette with 7μL of internal solution, tap the pipette to get rid of air bubbles, coat the pipette with molten dental wax (Sticky Wax; Kerr Corporation, Orange, CA) and mount it in the micromanipulator;
Turn the pipette overpressure on and lower the pipette slowly onto the preparation. Check pipette resistance in the amplifier and correct pipette offsets and capacitance with the respective amplifier controls;
Switch the superfusion to control ringer’s. To create a gigaseal between the pipette and the cell, slightly touch the tip of the electrode against the cell body and turn the pipette overpressure off while gently applying negative pressure to the electrode;
While sealed, choose “whole cell” mode in the amplifier and set the cell holding potential to -60 mV;
Choose a region of interest with the imaging software that encompasses the whole axon terminal, turn the bright field light off and, by briefly exposing (30 ms) the terminal to the 488 nm laser, find the right focal plane for TIRF imaging;
Break into the cell by using the “zap” command of the amplifier while applying slightly negative pressure to the pipette;
Correct for cell capacitance and series resistance, and then apply the voltage protocol of interest while imaging the movement of synaptic vesicles. It is at this stage important to have the voltage protocol synchronized to the camera frame rate. In our case, each frame is 30 ms long, so the voltage changes occur in multiples of this value (i.e., every 300 ms or 10 frames);
Wait at least 40 seconds between trials to allow for recovery;
To check the position of synaptic ribbons, take pictures while turning the 561 nm laser on.

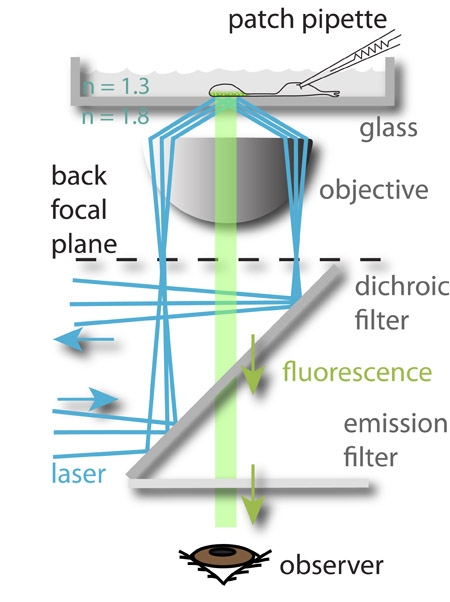
 **Figure 1: **The experimental setup. A 488 nm laser (blue) is focused to the periphery of the back focal plane of the objective and suffers total internal reflection when it reaches the glass-aqueous medium interface. The electromagnetic field generated by the reflected beam excites the fluorophore loaded into the synaptic vesicles closest to the bottom of the glass chamber, which then fluoresce (green). The fluorescent light is then guided to the observer’s eye (depicted) or to a CCD camera. The membrane potential of the imaged cells is controlled simultaneously by patch-clamping them. This approach allows the study of the relationship between incoming signals (the membrane voltage) and the neuronal output (exocytosis).


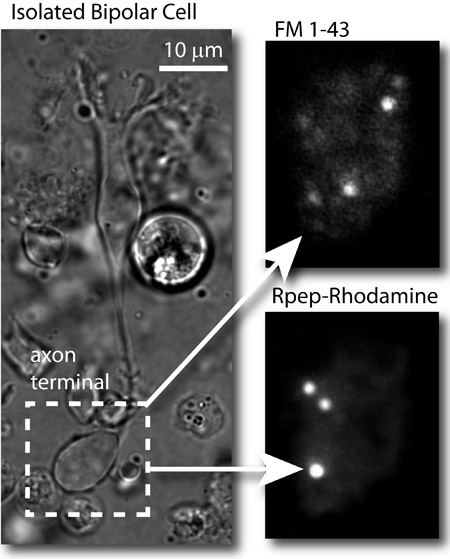
 **Figure 2: ** Typical results. Left: bright field image of an isolated goldfish bipolar cell. Upper right: TIRF image of the synaptic vesicles in the bipolar cell axon terminal loaded with FM 1-43® and imaged with the 488 nm laser (FM dye). Bottom right: image of the same terminal after patching the cell and imaging the axon terminal with the 561 nm laser. Synaptic ribbons are labeled by the rhodamine-based RIBEYE-binding peptide (Rpep_rhod).

**Table 1: **Specific Reagents and Equipment.

**Table:** 

Name	Type	Manufacturer	Catalog	Comment
-	Air Table	Newport Corporation	-	-
IX70	Inverted Microscope	Olympus	-	Equipped with a tungsten lamp for bright field and a lateral opening port for TIRF
TH4-100	Lamp Power Source	Olympus	-	-
FF498_581-Di01	Dichroic Filter	Semrock	-	-
NF01-405_488_568	Emission Filter	Semrock	-	-
Apo x100 O HR	Objective	Olympus	-	N.A. 1.65
RG630	Red Glass Filter	Schott	-	-
-	488 nm Laser	Coherent	-	Use at minimum power
-	Shutter	Uniblitz	-	-
VMM-D1	Shutter Driver	Uniblitz	-	-
-	561 nm Laser	Melles Griot	-	-
-	Shutter	Uniblitz	-	-
VMM-D3	Shutter Driver	Uniblitz	-	-
Perfusion Pressure Kit	Superfusion System	Automate Scientific	09-04	-
Perfusion Pen	Superfusion System	Automate Scientific	-	-
Valvelink 8	Superfusion System Controller	Automate Scientific	-	-
Cascade 512B	EM CCD Camera	Roper Scientific	-	-
Metamorph 7.1	Imaging Software	Molecular Devices	-	-
EPC-9	Patch Clamp Amplifier	HEKA Elektronik	-	-
Pulse	Amplifier Software	HEKA Elektronik	-	-
MP-285	Micromanipulator	Sutter Instrument	-	-
-	Electrode Holder	HEKA Elektronik	-	For 1.5 mm O.D. glass, 2 units
Kwik-Fil® TW150-3	Borosilicate Capillary Glass	WPI	-	Without filament
B150-86-10	Borosilicate Capillary Glass	Sutter Instrument	-	With filament
P-97	Microelectrode Puller	Sutter Instrument	-	Equipped with 3x3 box filament and environmental chamber
-	Pressure Vacuum Air Pump	Thomas Scientific	7893B05	Creates vacuum to remove liquids from chamber and overpressure for pipettes
MatLab R2008a	Analysis Software	MathWorks	-	-
353001	35 mm Plastic Culture Dishes	Falcon	-	-
-	High Refractive Index Glass	PlanOptik	-	Refractive index_488 nm_ = 1.78
Series M	High Refractive Index Liquid	Cargille Labs	-	Refractive index = 1.78
Sylgard 184	Silicon Elastomer Kit	Dow Corning	-	-
Glutathione	Tripeptide	EMD Chemicals		Free radical scavenger
Hyaluronidase	Enzyme	Sigma	H6254	Type V
L-cysteine	Amino Acid	Fluka	30090	Activates papain
Papain	Enzyme	Fluka	76220	From *Carica papaya*
Trolox®	Soluble Vitamin E	Sigma	56510	Free radical scavenger
ADVASEP-7	Sulfonated B-Cyclodextrin Derivative	Sigma	A3723	Reduces FM 1-43® background fluorescence
FM 1-43®	Fluorescent Dye	Invitrogen	T35356	“Special packaging”
Sticky Wax	Pipette Coating Agent	Kerr Corporation	-	Decreases pipette capacitance

**Table 2: **Physiological Solutions Used in This Study.

**Table:** 

Substance	Low Ca^2+^ Ringer’s	Control Ringer’s	High K^+^ Ringer’s	Internal Solution
NaCl	120 mM	120 mM	97.5 mM	-
KCl	2.5 mM	2.5 mM	25 mM	-
MgCl_2_	1 mM	1 mM	1 mM	4 mM
CaCl_2_	0.5 mM	2.5 mM	2.5 mM	-
HEPES	10 mM	10 mM	10 mM	10 mM
EGTA	0.75 mM	-	-	0.5 mM
Glucose	10 mM	10 mM	-	-
Glutathione	2 mM	2 mM	-	1 mM
CH_3_CsO_3_S*	-	-	-	100 mM
TEACl	-	-	-	10 mM
ATP-Mg	-	-	-	10 mM
GTP-Li	-	-	-	1 mM
Rpep-rhod**	-	-	-	5 mM
Volume	200 mL	100 mL	5 μL	100 μL

* Cesium methanesulfonate. ** RIBEYE-binding peptide: rhodamine+EQTVPVDLSVARDR-cooh (mw 1997.75).

## Discussion

The advantages of objective-type TIRF microscopy are that 1) it provides excellent optical sectioning by restricting excitation light to a narrow region within the focal plane of the objective, thereby minimizing out-of-focus light; 2) since light drops exponentially with distance, movement in a vertical direction can be monitored as a change in fluorescence intensity; 3) efficient light collection through the high numerical aperture objective^1,5^.

The main drawback of the technique is that it is limited to imaging events happening within 100& nm of the cell surface, which is roughly equivalent to an ultrathin section in electron microscopy. Therefore, visualization of these events depends critically on the cells being firmly adhered to the glass, on the presence of synaptic ribbons close to the patch of membrane adhered to the glass and on the successful loading of vesicles. Our protocol enables the loading of only 1-2% of the total number of vesicles within the bipolar cell synaptic terminal^2,6^. With that said, it is clear that there are much more events happening at the cell surface than the ones we are able to image.
